# Analgesic Effects of Duloxetine on Formalin-Induced Hyperalgesia and Its Underlying Mechanisms in the CeA

**DOI:** 10.3389/fphar.2018.00317

**Published:** 2018-04-10

**Authors:** Lie Zhang, Jun-Bin Yin, Wei Hu, Wen-Jun Zhao, Qing-Rong Fan, Zhi-Chun Qiu, Ming-Jie He, Tan Ding, Yan Sun, Alan D. Kaye, En-Ren Wang

**Affiliations:** ^1^Department of Neurosurgery, The First Affiliated Hospital of Chengdu Medical College, Chengdu, China; ^2^Department of Neurology, The 456th Hospital of PLA, Jinan, China; ^3^Department of Human Anatomy, The Fourth Military Medical University, Xi’an, China; ^4^Department of Orthopedics, Xijing Hospital, The Fourth Military Medical University, Xi’an, China; ^5^Cadet Bridge, The Fourth Military Medical University, Xi’an, China; ^6^Departments of Anesthesiology and Pharmacology, Louisiana State University School of Medicine, New Orleans, LA, United States

**Keywords:** central nucleus of amygdala (CeA), formalin model, pERK, duloxetine (DUL), hyperalgesia

## Abstract

In rodents, the amygdala has been proposed to serve as a key center for the nociceptive perception. Previous studies have shown that extracellular signal-regulated kinase (ERK) signaling cascade in the central nucleus of amygdala (CeA) played a functional role in inflammation-induced peripheral hypersensitivity. Duloxetine (DUL), a serotonin and noradrenaline reuptake inhibitor, produced analgesia on formalin-induced spontaneous pain behaviors. However, it is still unclear whether single DUL pretreatment influences formalin-induced hypersensitivity and what is the underlying mechanism. In the current study, we revealed that systemic pretreatment with DUL not only dose-dependently suppressed the spontaneous pain behaviors, but also relieved mechanical and thermal hypersensitivity induced by formalin hindpaw injection. Consistent with the analgesic effects of DUL on the pain behaviors, the expressions of Fos and pERK that were used to check the neuronal activities in the spinal cord and CeA were also dose-dependently reduced following DUL pretreatment. Meanwhile, no emotional aversive behaviors were observed at 24 h after formalin injection. The concentration of 5-HT in the CeA was correlated with the dose of DUL in a positive manner at 24 h after formalin injection. Direct injecting 5-HT into the CeA suppressed both the spontaneous pain behaviors and hyperalgesia induced by formalin injection. However, DUL did not have protective effects on the formalin-induced edema of hindpaw. In sum, the activation of CeA neurons may account for the transition from acute pain to long-term hyperalgesia after formalin injection. DUL may produce potent analgesic effects on the hyperalgesia and decrease the expressions of p-ERK through increasing the concentration of serotonin in the CeA.

## Introduction

Classically, the formalin test includes two well-identified phases of spontaneous pain behaviors, which is considered as a model of acute inflammatory pain (Wheeler-Aceto and Cowan, 1991; Rocha-Gonzalez et al., 2005; Sun et al., 2013). It is well accepted that the spontaneous pain response occurred immediately after formalin injection into the hindpaw or tail of rodent animals. The primary mechanism involved in this process was peripheral nervous system stimulation, namely, the direct activation of the peripheral transient receptor potential ankyrin (TRPA)-1 receptor (Adedoyin et al., 2010). Furthermore, formalin injection induced-secondary mechanical hyperalgesia was also observed after the acute phase (Wiertelak et al., 1994; Fu et al., 2000, 2001; Lin et al., 2007; Vierck et al., 2008; Yin et al., 2016). It has been demonstrated that the spontaneous pain response and secondary hyperalgesia were independent (Adedoyin et al., 2010). Therefore, the formalin test is a suitable model to investigate the transition from acute to chronic pain. Some studies revealed that formalin-induced long-term hyperalgesia was maintained by spinal dorsal horn (SDH) (Bravo-Hernandez et al., 2012) or descending facilitation from the rostral ventromedial medulla (RVM) (Ambriz-Tututi et al., 2011), however not sufficient to explain this hyperalgesia. In this study, we directed attention to the brain limbic system and tried to figure out the anatomic sites and underlying mechanisms involved in the transition from spontaneous pain to hyperalgesia induced by hindpaw formalin injection.

Neurons in the central nucleus of amygdala (CeA), a region of limbic system also called “nociceptive amygdala,” receive nociceptive information from the dorsal horn via afferent pathways relayed by the lateral parabrachial nucleus (LPB) (LPB-CeA pathway) (Dong et al., 2010). In rodents, this spinal cord-LPB-CeA neural pathway transmits most of the nociceptive information. Many studies have shown that the insular cortex and cingulate cortex are the brain areas which receive the CeA’s projections (Basbaum et al., 2009; Bliss et al., 2016). As the CeA was known to be involved in the acquisition and expression of emotion, this pathway was thought to play central roles in both inducing and maintaining affective aspects of pain responses. It has been demonstrated that excitatory synaptic transmissions were potentiated on the LPB-CeA pathway in some inflammatory pain models, such as arthritic and muscle pain models (Neugebauer et al., 2009; Cheng et al., 2011). Moreover, the excitability of CeA also increased in some chronic pain models, such as spinal nerve ligation (SNL) pain model (Nakao et al., 2012). Therefore, we assumed that neuroplasticity in the CeA plays a pivotal role in the transition from acute to chronic pain and the initiation of long-term hyperalgesia induced by formalin injection.

Serotonin depletion has long-term effects on the functional activity of the nociceptive system and there was an important role of 5-HT in mediating the effects of stress on pain sensitivity in the formalin test (Butkevich et al., 2005a,b). The increased nociception in prenatally stressed 7-day-old pups might be associated with the decrease in the intensity of serotonin-like immunoreactivity and density of serotonergic cells (Butkevich et al., 2006). Meanwhile, 5-HT_2C_ receptor knockdown in the amygdala inhibited neuropathic-pain-related plasticity and behaviors (Ji et al., 2017). Duloxetine (DUL) was primarily administered to treat depressive disorder through increasing the concentration of serotonin/noradrenaline in the synapse (Cipriani et al., 2009; Mancini et al., 2012). Further studies have demonstrated its wide analgesia on multi-types of pain, including fibromyalgia (Hauser et al., 2009), diabetic neuropathy (Raskin et al., 2006), functional chest pain (Wang et al., 2012), osteoarthritic pain (Citrome and Weiss-Citrome, 2012) and non-organic orofacial pain (Nagashima et al., 2012). However, other specifical underlying mechanisms of DUL as pain killer are still unknown. We hypothesized here that CeA relates with the transition from acute to chronic pain induced by formalin injection. DUL can exert analgesic effects on formalin-induced long-term hyperalgesia and regulating the activation of extracellular signal-regulated kinases (ERK) in the CeA through modifying the concentration of CeA 5-HT.

## Materials and Methods

### Animals and Drugs

Male C57BL/6 mice (about 10 weeks old) were housed in a temperature-controlled environment on a 12-h light/dark cycle with access to food and water *ad libitum*. The mice would be handled before doing any operation. To reduce the suffering of mice before anesthesia, all the operations must be gentle and quick at a comfortable environment. All experimental protocols were in accordance with the ethical guidelines and received prior approval from the Animal Use and Care Committee for Research and Education of the Fourth Military Medical University (Xi’an, China). Formalin solution was bought from Si’chuan Xi’long Chemical Co., Ltd. (Chengdu, China). DUL (Eli Lilly Company, United States) was purchased and freshly dissolved in sterile saline, filtered before use and delivered intraperitoneally (*i.p.*).

### Experimental Design

According to our pilot experiment, the behavioral features of mice receiving *s.c.* saline injection were similar to those of naïve mice, thus the data obtained from the naïve mice were not included in the current study. To reduce the bias introduced by the batch difference of animals, as well as to better control and compare the results, we used separate vehicle groups (*s.c.* saline injection) for this experiment.

We aimed to establish the dose-effect curve for DUL on the formalin induced pain responses. After 1 week acclimation, the animals were randomly assigned to one of the following groups (9 mice in each group): mice receiving *i.p.* injection with saline (Veh group), 1 mg/kg of DUL (DUL 1 mg/kg group), 3 mg/kg of DUL (DUL 3 mg/kg group), 10 mg/kg of DUL (DUL 10 mg/kg group), 30 mg/kg of DUL (DUL 30 mg/kg group) then followed by 25 μl of 5% formalin *s.c.* injected into the plantar surface of the hindpaw 1 h later. The animals from all groups were video-recorded for the later analysis during the 1 h time window. And the mechanical threshold and thermal latency of the injected paw were tested at 0/1/3/24 h after formalin injection. After formalin injection 2 h, three mice in each group were perfused for the immunohistochemical staining of FOS and phosphorylation ERK (p-ERK) in the SDH and CeA; after formalin injection 24 h, other 3 mice in each group were also perfused for the staining of FOS and p-ERK in the SDH and CeA.

We also tested the effects of DUL after the mechanical and thermal hyperalgesia were established. At 24 h after formalin injection, the above doses of DUL were administered and the mechanical and thermal hyperalgesia were tested 1 h later (6 mice in each group).

### Formalin Test

The formalin test was established to observe the spontaneous pain responses (flinching or licking the injected hind paw). Mice were brought to the lab and placed in the test chamber for 20 min each day for 7 days. After the mice’s acclimation to the testing chamber for about 20 min, 25 μl of the 5% formalin solution (dissolved in saline) was *s.c.* injected into the plantar surface of the left hindpaw using a microsyringe (Hamilton co. Reno, NV, United States) attached to a 30-G needle. After formalin administration, the mice were returned to the test chamber and the video-recordings were performed for 60 min, as described below.

All the following behavioral recording was conducted by a tester blinded to the experimental condition. A sound-attenuated clear perspex testing chamber (25 × 25 × 40 cm) was fitted with an inverted video camera to record video for offline behavioral analysis. A trained observer, who was blinded to different groups, conducted the behavioral analysis of the video recordings to determine the spontaneous pain responses induced by formalin. The pain behaviors were manually recorded by retrieving behaviors from the recorded videos. As previous studies have suggested (Dubuisson and Dennis, 1977; Saddi and Abbott, 2000; Akbari et al., 2013), the behavioral rating criteria were as follows: (1) no pain: normal weight bearing on the injected paw; (2) favoring: injected paw resting lightly on floor or limping; (3) lifting: elevation of the injected paw; (4) licking: licking or biting the injected paw. Weighted pain scores were used to evaluate the spontaneous pain behaviors, in which no pain is weighted 0, favoring 1, lifting 2, and licking 3. The pain scores was 0 × normal + 1 × favoring + 2 × lifting + 3 × licking.

### Measurements of Mechanical Threshold and Thermal Latency

Experiments were performed on the mice of each group, respectively. To quantify the mechanical sensitivity of the hindpaw, animals were placed in individual plastic boxes and allowed to acclimate for 30 min. The method was described in our previous studies (Yin et al., 2016; Zhao et al., 2017). A series of calibrated von Frey filaments (Stoelting, Kiel, WI, United States) were applied to the plantar surface of the hindpaw (ranging from 0.02 g to 10.0 g) with a sufficient force to bend the filaments for 5 s or until paw withdraw. In the presence of a response, the filament of the next lower force was applied. In the absence of a response, the filament of the next greater force was applied. A sharp withdrawal of the paw indicates a positive response. Each filament was applied five times and the minimal value which caused at least three responses was recorded as the paw withdrawal thresholds. The stimulus was stopped if the threshold exceeded 10.0 g force (cutoff value). Assessment were made before formalin injection as a baseline.

Thermal hyperalgesia was investigated by using Hargreaves test (Wu et al., 2014; Lin et al., 2017). Paw withdrawal in response to noxious thermal stimuli was assessed using a radiant heat source (8 V, 50 W; Ugo Basile, Comerio, VA, Italy). Mice were placed in plastic boxes on a glass plate for at least 30 min before testing. The time from initiation of the light beam to paw withdrawal was recorded as paw withdrawal latency. The intensity of the beam was set to produce a basal latency of approximate 4–6 s. A cut-off time of 15 s was set to prevent skin damage. Three measures of latency were taken in the same hindpaw and averaged.

### Self-Grooming Behaviors

Spontaneous self-grooming behaviors was investigated as previously described (Kalueff and Tuohimaa, 2005; Dhamne et al., 2017; Fujita et al., 2017). Each mouse was placed individually into a standard mouse cage (46 cm length × 23.5 cm wide × 20 cm high). Cages were empty to eliminate digging in the bedding, which is a potentially competing behavior. A front-mounted camera was placed at about 1 m from the cages to record the sessions, which were videotaped for 60 min. Each mice was scored for cumulative time spent on grooming all the body regions (i.e., forepaws, nose/face, head, body, hind legs/tail/genitals) and the number of bounts during the 60 min test session. If the interval between two bounts was >5 s, then they were counted as separate bounts.

### Open Field (OP) Test

The testing room remained quiet and dusk with indirect lighting during the experiment. Mice were softly placed at the center of the testing chamber [47 cm (W) × 47 cm (H) × 47 cm (D)] after 1 h acclimation to the testing room. The automated analyzing system (Shanghai Mobile datum Information Technology Co., Ltd.) recorded the track of mice for 15 min (Sun et al., 2013; Zhai et al., 2016). The total distance and time percentage in the central area were evaluated to represent the locomotion and anxiety/depression levels of mice.

### Elevated Plus Maze (EPM) Test

The mice were placed at the central area of EPM, which constituted with two closed arms (CA, 50 × 10 cm), two open arms (OA, 50 × 10 cm) and a central area (10 × 10 cm). The bottom of the EPM was 50 cm above ground. The automated analyzing system recorded the video for 5 min. The numbers of the mice entering each arms and the amount of time the mice spent on each arm was analyzed by two investigators blinded to the experiment. Four paws of the mice onto the open arm were recorded as an entry. OA entry time % and OA entries % were scored as described previously (Sun et al., 2013; Zhai et al., 2016).

### Cannula Implantation

For microinjection of 5-HT into the CeA, the mice were initially anesthetized with sodium pentobarbital (50 mg/kg, i.p.). A 5.0 mm length guide cannula (6202, OD 0.56 × ID 0.38 mm, RWD, Shenzhen, China) was stereotaxically implanted, aimed at the CeA (AP: -1.46 mm; ML: +2.7 mm; DV: +4.2 mm), fixed to the skull with bone screws, super glue, dental cement, and a dummy cannula was inserted into the guide cannula. After guide cannula implantation and a 1 week recovery, mice were applied to test for pain behaviors.

### Measurement of Serotonin Levels

Mice were sacrificed after deep anesthesia by using pentobarbital (100 mg/kg, i.p.). Brains were removed and sectioned into 1 mm thickness coronal sections using an acrylic brain matrix on the ice. From the two appropriate sections based on the brain atlas, amygdala punches were obtained using a custom-made 0.5 mm punch tool. The CeA (both the medial and lateral sub nuclei) located close to the inferior segment of external capsule on the medial side. To determine the serotonin level, the CeA in each group was homogenated with 1 ml of perchloric acid containing 0.1% cysteine, then centrifuged at 10,000 × *g* for 20 min at 4°C, and the supernatant was collected and stored at -70°C. The levels of serotonin were measured with a commercially available ELISA according to the manufacturer’s instructions (LDN, Nordhorn, Germany).

### Formalin-Induced Paw Edema

After 25 μl of the 5% formalin solution was *s.c.* injected into the plantar surface of the left hindpaw, the volume (ml) of this injected hindpaw was measured using a plethysmograph at 0/1/3/24 h after formalin injection. The increase of paw volume in each groups was calculated. And we also take pictures of the hindpaw at each timepoints.

### Immunohistochemistry Staining

After deep anesthesia by using pentobarbital (100 mg/kg, i.p.), mice were perfused intracardially with 20 ml phosphate-buffered saline (PBS, pH = 7.4) and subsequently with 50 ml 4% paraformaldehyde in 0.1 M phosphate buffer (PB, pH = 7.4). Brains and spinal cords were removed and post-fixed in the same fixative overnight. Then all tissues were transferred to 30% sucrose in 0.1 M PB at 4°C at least 24 h for cryoprotection. Brains and spinal cords were mounted in block and cut on a cryostat (Leica CM1800, Heidelberg, Germany) at the thickness of 30 μm at -20°C. Sections were collected serially into dishes containing 0.01 M PBS. The sections containing the cannula injection sites were stained with cresyl violet.

All sections used for the immunofluorescent staining were blocked with 10% normal donkey serum (NDS) in 0.01 M PBS with 0.3% Triton X-100 for 1 h at room temperature and then incubated overnight at 4°C with a mixture of rabbit anti-p-ERK (1:200; 4370; Cell Signaling Technology, Beverly, MA, United States) and mouse anti-Fos (1:500; ab11959; Abcam, Cambridge, MA, United States) antibodies in PBS containing 1% NDS and 0.3% Triton X-100. After 3 rinses in PBS, the sections were incubated with Alexa488 donkey anti-rabbit IgG (1:500; A21201; Invitrogen, Carlsbad, CA, United States) and Alexa594 donkey anti-mouse IgG (1:500; A21203; Invitrogen) for 4 h at 4°C. After three washes in PBS, the sections were mounted and coverslipped on microscope slides. These sections were observed and images were captured under confocal laser scanning microscope (FV1000, Olympus, Tokyo, Japan) with appropriate filters for Alexa488 (excitation 492 nm, emission 520 nm), or Alexa594 (excitation 590 nm, emission 618 nm).

The p-ERK immunostaining in the CeA after different dose DUL treatment was performed by using the ABC method (Wang et al., 2015; Zhao et al., 2017). Briefly, after incubation with 3% H_2_O_2_ for 10 min, sections were washed with 0.01 M PBS and then incubated with 10% NDS for 30 min. Sections containing the CeA regions were sequentially incubated with the following: rabbit anti-p-ERK (1:200); biotinylated goat anti-rabbit IgG antibody (1:200; Cell Signaling Technology); and avidin-biotin-peroxidase complex (ABC) (ABC Elite Kit; 1:200; Vector Laboratories). They were then visualized with diaminobenzidine (DAB) chromogen. Sections were then observed under a light microscope (AH-3; Olympus, Tokyo, Japan).

The specificities of the immunohistochemistry staining were tested on the sections from the other dishes by omitting the primary specific antibodies. The other procedures and antibodies were the same with the above staining experiments. No immunoreactive products were found on the sections. The numbers of Fos and p-ERK in the sections were counted by an observer blinded to the experimental conditions.

### Dose-Effect Curve and ED_50_ Calculation

The dosages of DUL were transformed into logarithm dose and the non-line fitness was performed so as to build the dose-effect curve. Based on the dose-effect cure, the ED_50_s of DUL were calculated. The reliability of ED_50_ calculated from a specific dose-effect curve can be evaluated by the slope factor returned by the GraphPad Prism version 5.01 for Windows (GraphPad Software, San Diego, CA, United States)^1^.

### Statistical Analysis

The results were expressed as mean value ± standard error of the mean (SEM). In the formalin test, when comparing the pain responses, data from the first phase, the second phase and secondary pain were considered independently. The AUC of individual animal for formalin pain response curves were group pooled and One-way ANOVA with Dunnett’s *post hoc* test was performed using GraphPad Prism version 5.01 for Windows.

## Results

### DUL Dose-Dependently Suppressed Formalin-Induced Spontaneous Pain Responses

**FIGURE 1:**
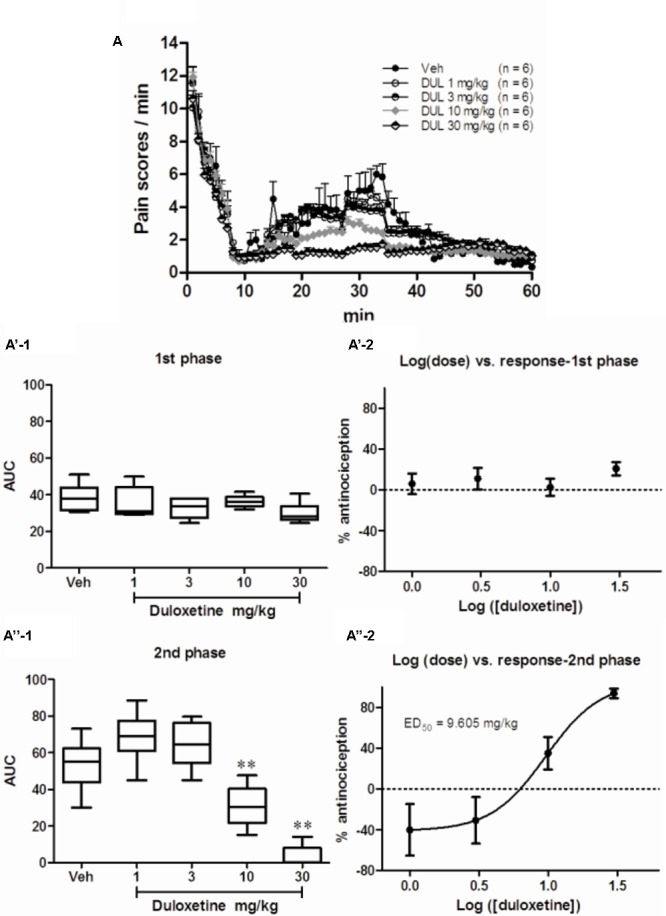
The analgesic effects of DUL on the first and second phase of spontaneous pain responses after formalin injection. Spontaneous pain behavior indicated by pain scores during 60 min after subcutaneous formalin injection from different groups were shown in **(A)**. The areas under curve for different groups were calculated to perform statistical analysis on first **(A’-1)** and second **(A”-1)** phases. The log (dose)-effect curves for DUL’s analgesic effects were shown in **(A’-2)** (first phase) or **(A”-2)** (second phase). ^∗∗^*P* < 0.01, one-way ANOVA, Dunnett’s *post hoc* test, *n* = 6 in each group.

**FIGURE 2:**
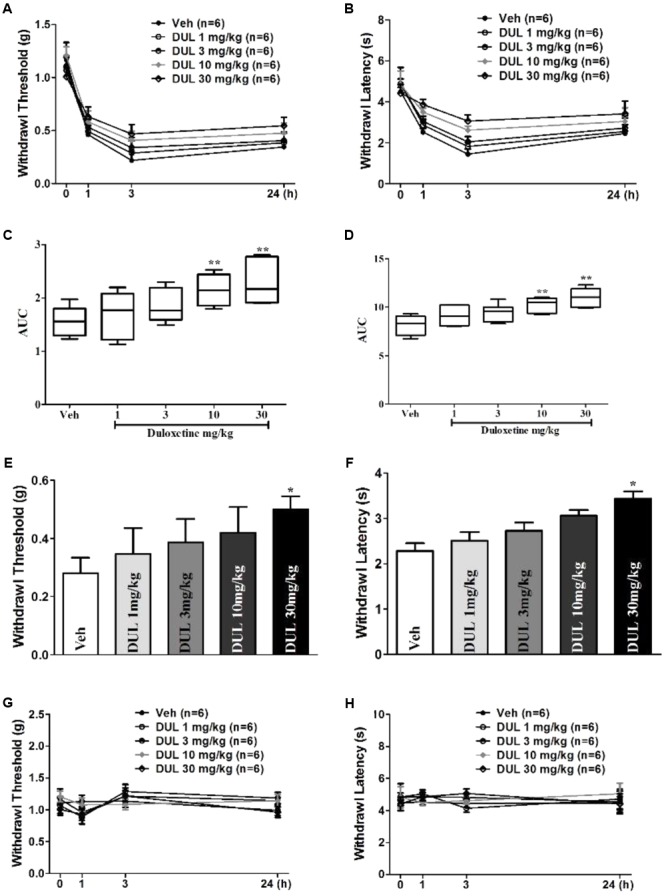
The analgesic effects of DUL on the mechanical and thermal hyperalgesia after formalin injection. The mechanical thresholds **(A)** and thermal latencies **(B)** of hindpaw after formalin injection were tested in different groups. The areas under curve for different groups were calculated to perform statistical analysis on mechanical thresholds **(C)** and thermal latencies **(D)**. At 24 h after formalin injection, the mechanical thresholds **(E)** and thermal latencies **(F)** of hindpaw were tested following different dose DUL administration 1 h later. The DUL treatment has no significant influence on the mechanical thresholds **(G)** and thermal latencies **(H)** of mice without formalin injection. ^∗^*P* < 0.05, ^∗∗^*P* < 0.01, one-way ANOVA, Dunnett’s *post hoc* test, *n* = 6 in each group.

As observed in our previous study, injection of 5% formalin *s.c.* into the plantar surface of the hindpaw produced biphasic pain-related behaviors (**Figure 1A**). The first transient phase lasted for the first 10 min post injection and was followed by the second prolonged phase from 15 to 60 min. Pretreatment with DUL (*i.p.*) significantly reduced pain scores at the second but not the first phase. There was no group difference on pain scores at the first phase [**Figures 1A,A’-1**; one way ANOVA, *F*(4,29) = 0.1865, *P* = 0.943]. Given the negative effect of DUL on pain scores at the first phase, the ED_50_ value could not be retrieved based on the log (dose) *vs.* response curve (**Figure 1A’-2**). While, there existed a significant group difference on pain scores at the second phase [**Figures 1A,A”-1**; one way ANOVA, *F*(4,29) = 12.39, *P* < 0.05]. Dunnett’s *post hoc* test also revealed group difference between DUL 10 mg/kg (*P* < 0.05) or DUL 30 mg/kg (*P* < 0.05) and vehicle treatment. The ED_50_ of DUL on pain scores at the second phase was 9.605 mg/kg, which was calculated based on the log (dose) *vs.* response curve (**Figure 1A”-2**).

### DUL Dose-Dependently Alleviated Formalin-Induced Hyperalgesia

To further investigate whether single DUL treatment have long-term effects, formalin-induced mechanical and thermal hyperalgesia were tested. Von Frey filaments experiment showed a significant group difference on the withdrawal thresholds of the hindpaw following formalin injection 24 h [**Figures 2A,C**; one way ANOVA, *F*(4,29) = 14.56, *P* < 0.05]. Dunnett’s *post hoc* test revealed group differences between DUL 10 mg/kg (*P* < 0.01) or DUL 30 mg/kg (*P* < 0.01) and vehicle treatments. Hargreaves test also showed the similar analgesic effects of DUL. There was a significant group difference on the withdrawal latencies of the hindpaw [**Figures 2B,D**; one way ANOVA, *F*(4,29) = 8.46, *P* < 0.05]. Dunnett’s *post hoc* test revealed group differences between DUL 10 mg/kg (*P* < 0.01) or DUL 30 mg/kg (*P* < 0.01) and vehicle treatments.

These results indicate the important role of transition from acute to chronic pain on the hyperalgesia establishment. DUL has potent analgesic effects by disrupting this transition. We subsequently investigated the analgesic effects of DUL after hyperalgesia were established. After formalin injection 24 h, we administered DUL and tested the mechanical and thermal hyperalgesia 1 h later. What is different, only DUL 30 mg/kg had analgesic effects on the mechanical threshold (**Figure 2E**, *P* < 0.05) and thermal latency (**Figure 2F**, *P* < 0.05). The administration of saline or DUL had no influence on the mechanical threshold (**Figure 2G**) and thermal latency (**Figure 2H**) of the normal mice without formalin injection. DUL directly produced analgesic effects, while did not change the normal mechanical threshold or thermal latency.

### Formalin Injection Increased the p-ERK and Fos Expressions in the SDH and CeA

**FIGURE 3:**
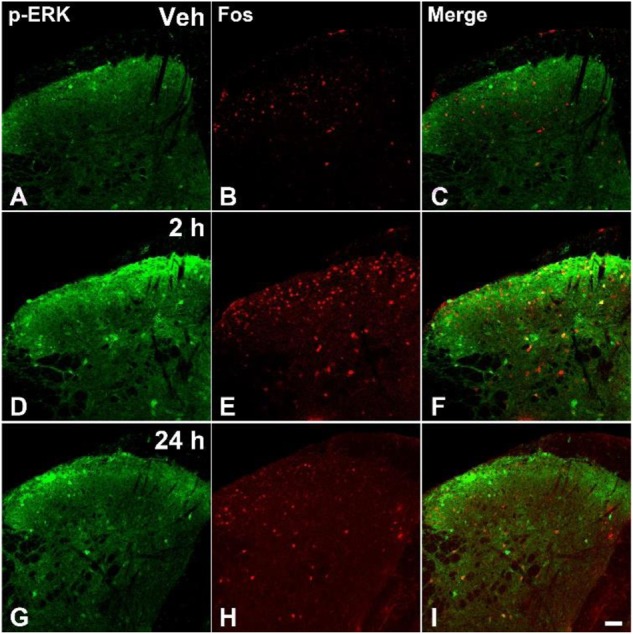
The immunofluorescent staining of Fos and p-ERK in the SDH after formalin injection. The double-staining of pERK (green) and Fos (Red) was conducted in the ipsilateral SDH after vehicle injection **(A–C)**, formalin injection 2 h **(D–F)**, and formalin injection 24 h **(G–I)**. Scale bar = 50 μm in **(I)** (suitable for **A–H**).

**FIGURE 4:**
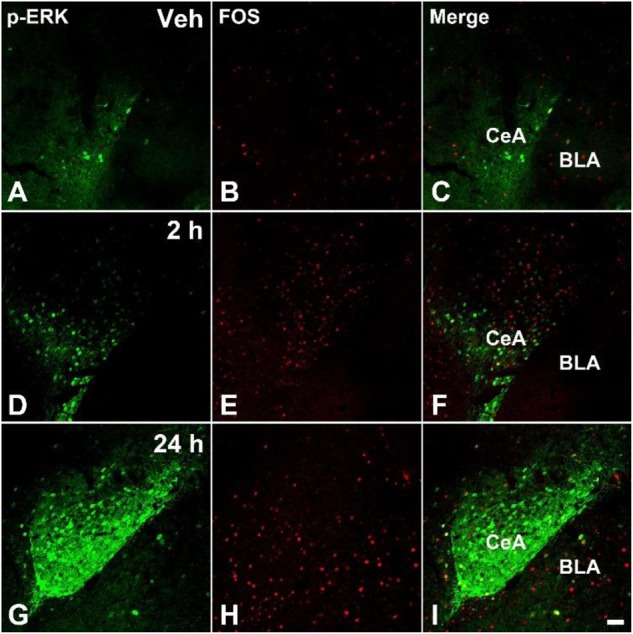
The immunofluorescent staining of Fos and p-ERK in the CeA after formalin injection. The double-staining of pERK (green) and Fos (Red) was conducted in the contralateral CeA after vehicle injection **(A–C)**, formalin injection 2 h **(D–F)**, and formalin injection 24 h **(G–I)**. Scale bar = 50 μm in **(I)** (suitable for **A–H**).

**FIGURE 5:**
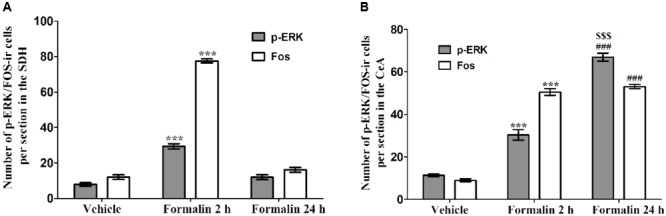
The expressions of p-ERK and Fos in the ipsilateral SDH and contralateral CeA after formalin injection. The expressions of p-ERK and Fos in the ipsilateral SDH after formalin injection **(A)**. The expressions of p-ERK and Fos in the contralateral CeA after formalin injection **(B)**. ^∗∗∗^*P* < 0.001, formalin injection 2 h group compared with vehicle group; ^###^*P* < 0.001, formalin injection 24 h group compared with vehicle group; ^$$$^*P* < 0.001, formalin injection 24 h group compared with formalin injection 2 h group; one-way ANOVA, Turkey’s *post hoc* test, *n* = 18 sections in each group.

The acute pain behaviors and long-term hyperalgesia induced by formalin injection, may be produced by different mechanisms in the nervous system. The double staining of p-ERK and Fos was used to check the activation of neurons in the SDH and CeA. There were a few p-ERK-ir or Fos-ir neurons observed in the SDH (**Figures 3A–C**) and CeA (**Figures 4A–C**) of the vehicle treated mice. The expressions of p-ERK and Fos presented temporal changes in the ipsilateral superficial layers of SDH (**Figures 3D–I**) and in the contralateral CeA (**Figures 4D–I**) after formalin injection. The expressions of p-ERK [**Figure 5A**; one way ANOVA, *F*(2,51) = 77.03, *P* < 0.001; Turkey’s *post hoc* test: vehicle group *vs.* 2 h group, *P* < 0.001, vehicle group *vs.* 24 h group, *P* > 0.05] and Fos [one way ANOVA, *F*(2,51) = 806.4, *P* < 0.001; Turkey’s *post hoc* test: vehicle group *vs.* 2 h group, *P* < 0.001, vehicle group *vs.* 24 h group, *P* > 0.05] reached its peak at 2 h and gradually reduced at 24 h in the SDH. The expressions of p-ERK and Fos increased gradually in the CeA [**Figure 5B**; p-ERK: one way ANOVA, *F*(2,51) = 238.9, *P* < 0.001; Fos: one way ANOVA, *F*(2,51) = 463.1, *P* < 0.001]. Turkey’s *post hoc* test revealed differences between vehicle group and 2 h group on p-ERK (*P* < 0.001) and Fos (*P* < 0.001) expressions. Meanwhile, there were significant increases on p-ERK (*P* < 0.001) and Fos (*P* < 0.001) expressions in the 24 h group compared with vehicle group. Moreover, the expressions of p-ERK (*P* < 0.001) in the 24 h group were higher than those in the 2 h group. This demonstrates that the transition from formalin-induced acute pain to long-term hyperalgesia may be related to the activation of the CeA, but not the SDH.

### DUL Dose-Dependently Inhibited p-ERK and Fos Expressions in the SDH and CeA

**FIGURE 6:**
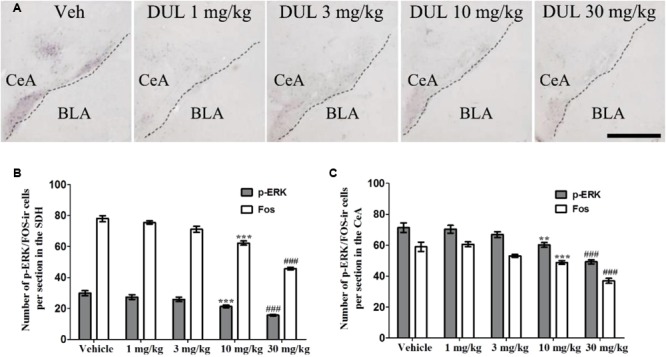
DUL dose-dependently inhibited the p-ERK and Fos expressions in the ipsilateral SDH and contralateral CeA. Representative immunostaining images showed the p-ERK expressions in the contralateral CeA after formalin injection 24 h, in different treatment groups **(A)**. DUL dose-dependently inhibited the p-ERK and Fos expressions in the ipsilateral SDH after formalin injection 2 h **(B)**. DUL dose-dependently inhibited the p-ERK and Fos expressions in the contralateral CeA after formalin injection 24 h **(C)**. Scale Bar = 200 μm in A. ^∗∗^*P* < 0.01, ^∗∗∗^*P* < 0.001, DUL 10 mg/kg group compared with vehicle group; ^###^*P* < 0.001, DUL 30 mg/kg group compared with vehicle group; one-way ANOVA, Turkey’s *post hoc* test, *n* = 18 sections in each group.

Next, we analyzed the effects of DUL on the formalin-induced p-ERK and Fos expressions in the SDH and CeA. Representative immunostaining images showed the p-ERK expressions in the CeA after formalin injection 24 h, in different treatment groups (**Figure 6A**). Notably, at 2 h after formalin injection, the activation of p-ERK and Fos in the SDH were significantly inhibited by DUL in a dose-dependent manner [**Figure 6B**, p-ERK: one way ANOVA, *F*(4,85) = 20.11, *P* < 0.001; Fos: one way ANOVA, *F*(4,85) = 73.66, *P* < 0.001]. Turkey’s *post hoc* test also revealed group differences between vehicle group and DUL 10 mg/kg group (*P* < 0.001) or DUL 30 mg/kg group (*P* < 0.001) on p-ERK expressions. Meanwhile, there were significant decreases on Fos expressions in DUL 10 mg/kg group (*P* < 0.001) or DUL 30 mg/kg group (*P* < 0.001) compared with vehicle group. At 24 h after formalin injection, the expressions of p-ERK and Fos in the CeA were also significantly inhibited by DUL in a dose-dependent manner [**Figure 6C**, p-ERK: one way ANOVA, *F*(4,85) = 18.33, *P* < 0.001; Fos: one way ANOVA, *F*(4,85) = 26.69, *P* < 0.001]. Turkey’s *post hoc* test also revealed group differences between vehicle group and DUL 10 mg/kg group (*P* < 0.01) or DUL 30 mg/kg group (*P* < 0.001) on p-ERK expressions. Meanwhile, there were significant decreases on Fos expressions in DUL 10 mg/kg group (*P* < 0.001) or DUL 30 mg/kg group (*P* < 0.001) compared with vehicle group.

### DUL Had No Influence on the Limbic-Related Behaviors

**FIGURE 7:**
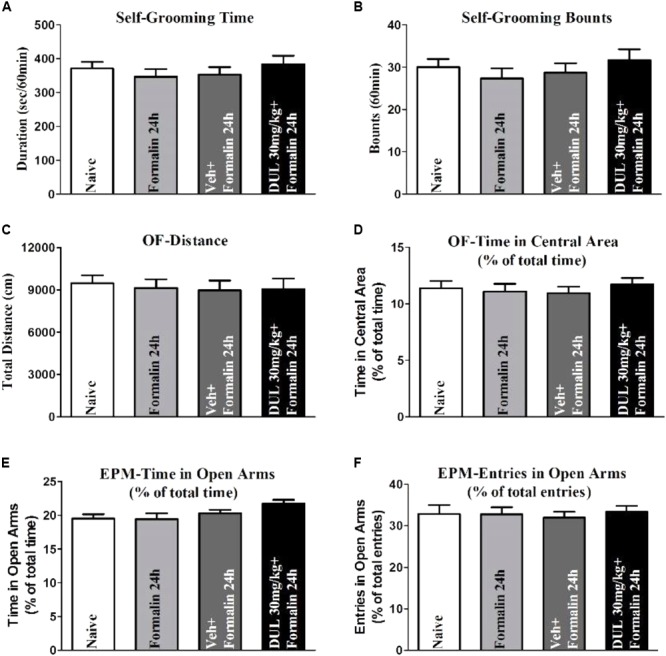
The DUL administration has no effects on the limbic-related behaviors after formalin injection 24 h. Formalin injection 24 h and DUL 30 mg/kg have no influence on the self-grooming time **(A)** and bounts **(B)**. There were no significant differences on the total distance **(C)** and time in the central area **(D)** in the OF test among different groups. Similarly, there were also no significant differences on the time **(E)** and entries **(F)** in open arms in the EPM test among different groups. One-way ANOVA, *n* = 6 in each group.

Actually, CeA is also an important brain area involved into limbic-related behaviors, which includes depression, anxiety, and fear memory. While, these limbic-related behaviors also affected nociceptive information perception. Therefore, we would like to check whether DUL modifies the limbic-related behaviors after formalin injection 24 h. The self-grooming time and bounts were not changed after formalin injection 24 h (**Figures 7A,B**). There was no significant difference between vehicle group and DUL 30 mg/kg group on the self-grooming time (*P* > 0.05) and bounts (*P* > 0.05). The total distance and time percentage spent in the central area in the OF test were also not changed after formalin injection 24 h (**Figures 7C,D**). There was no significant difference between vehicle group and DUL 30 mg/kg group on the total distance (*P* > 0.05) and time percentage spent in the central area (*P* > 0.05). The time and entries percentages spent in the open arms in the EPM test were also not affected after formalin injection 24 h (**Figures 7E,F**). There was no significant difference between vehicle group and DUL 30 mg/kg group on the time (*P* > 0.05) and entries (*P* > 0.05) percentages spent in the open arms. These results indicate that there were no limbic-related behaviors after formalin injection 24 h and DUL had no influences on them.

### Involvement of 5-HT in the CeA on the Analgesic Effects of DUL

**FIGURE 8:**
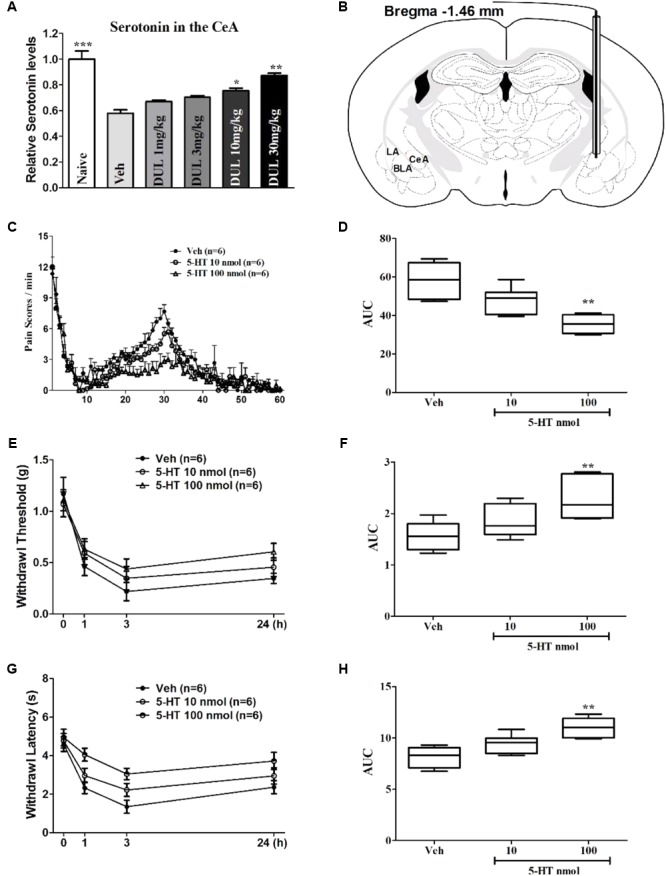
The roles of 5-HT in the CeA on the formalin injection-induced spontaneous pain response and secondary hyperalgesia. DUL dose-dependently lightened the formalin injection-induced reductions of serotonin levels in the contralateral CeA **(A)**. The diagram image in **(B)** showed that the cannular was implanted into the CeA (Bregma –1.46 mm). Local infusion 5-HT dose-dependently decreased the formalin injection-induced spontaneous pain responses **(C,D)**. The reductions of mechanical **(E,F)** and thermal **(G,H)** hyperalgesia after formalin injection were dose-dependently alleviated with CeA 5-HT injection. ^∗^*P* < 0.05, ^∗∗^*P* < 0.01, One-way ANOVA, Dunnett’s *post hoc* test, *n* = 6 in each group.

To better understand the underlying mechanisms for the analgesic effects of DUL on the formalin-induced pain behaviors, we tested the concentration of 5-HT in the CeA and injected 5-HT into the CeA directly to check its effects on pain behaviors. Firstly, formalin hindpaw injection reduced the concentration of 5-HT in the contralateral CeA by using Elisa method (**Figure 8A**, *P* < 0.001). Dunnett’s *post hoc* test also revealed group differences between DUL 10 mg/kg (*P* < 0.05) or DUL 30 mg/kg (*P* < 0.01) and vehicle treatments. The mice were received cannula implantation on the CeA and then used to do the formalin test (**Figure 8B** and Supplementary Figure S1). The spontaneous pain behaviors were checked after local infusion 5-HT (10 and 100 nmol) into the CeA 1 h (**Figure 8C**). There was a significant difference between 5-HT 100 nmol and vehicle treatment group (**Figure 8D**, *P* < 0.01). Meanwhile, we also investigated the effects of 5-HT CeA injection on the mechanical threshold (**Figure 8E**) and thermal latency (**Figure 8G**). Of particular interest, local infusion 5-HT (100 nmol) into the CeA significantly produced analgesic effects on the mechanical (**Figure 8F**, *P* < 0.01) and thermal (**Figure 8H**, *P* < 0.01) hyperalgesia. These results indicate that DUL exerted obvious analgesic effects through enhancing the serotonin levels in the CeA after formalin injection.

### DUL Had No Protective Effects on Increased Paw Edema

**FIGURE 9:**
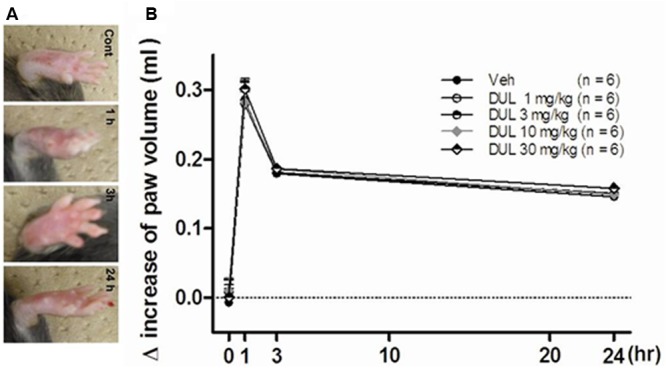
**(A)** The representative photographs showed the paw edema induced by formalin injection at 1, 3, and 24 h. **(B)** The effects of different dose DUL (1, 3, 10, and 30 mg/kg) on hindpaw edema induced by formalin injection. There was no significant difference among these groups. One-way ANOVA, Dunnett’s *post hoc* test, *n* = 6 in each group.

As observed in **Figure 9A**, the edema induced by formalin could be observed at 1 and 3 h after formalin injection, then the edema decreased at 24 h after formalin injection. There were no significant group differences on the increased paw volume at 1, 3, and 24 h in comparison with Veh group [**Figure 9B**; one way ANOVA, *F*(4,29) = 3.27, *P* > 0.05]. Dunnett’s *post hoc* test also revealed no significant differences between 1, 3, 10, or 30 mg/kg and vehicle treatment groups (*P* > 0.05).

## Discussion

Formalin (5%) hindpaw *s.c.* injection has been demonstrated to induce acute spontaneous pain behaviors (0–1 h), and subsequently long-term (1–24 h) secondary hyperalgesia in the ipsilateral hindpaw. Secondary nociceptive behaviors were observed in various experiments, which were similar using 10% (Cadet et al., 1993), 5% (Fu et al., 2000, 2001; Wu et al., 2001; Vierck et al., 2008), 1% (Ambriz-Tututi et al., 2009) and 0.5% formalin (Jolivalt et al., 2006). Furthermore, the mechanical and thermal hyperalgesia were also observed at 24 h after formalin injection. The expressions of FOS and p-ERK in the SDH might be the underlying mechanism for the acute spontaneous pain responses. Whereas, the activation of CeA neurons might be the reason for the transition from formalin-induced spontaneous pain to long-term hyperalgesia, which was indicated by the increase expressions of p-ERK in the CeA. These analgesic effects of DUL was related to the levels of 5-HT in the CeA. The edema induced by the formalin injection did not change a lot following different dosage of DUL. Our results suggest that the activations of neurons in different nucleus account for different stage of formalin hindpaw injection-induced pain behaviors.

### The Analgesic Effects of DUL on the Formalin-Induced Spontaneous Pain

DUL is one of serotonin/noradrenaline reuptake inhibitors (SNRI), which increases the concentration of serotonin/noradrenaline in the synapse. Its affinities for muscarinic, α1 adrenergic, and histamine H1 receptors are weaker than those of tricyclic antidepressants (TCAs), which imply its weaker adverse effects and potential application in clinic (Bymaster et al., 2001). DUL has been demonstrated widely analgesic efficacy for fibromyalgia (Hauser et al., 2009), diabetic neuropathy (Raskin et al., 2006), functional chest pain (Wang et al., 2012), osteoarthritic pain (Citrome and Weiss-Citrome, 2012) and non-organic orofacial pain (Nagashima et al., 2012) in clinic. Our previous study suggests that DUL pretreatment mainly attenuated the second phase of formalin-induced spontaneous pain responses more than the first phase *in vivo* (Sun et al., 2013). And this analgesic effect may be preferentially mediated by spinal not supra-spinal mechanisms. The current study also showed the effects of DUL on spontaneous pain behaviors induced by formalin. Furthermore, we observed the mechanism involved in this process was due to inhibit the expressions of p-ERK and FOS in the SDH, but not the peripheral inflammatory reaction indicated by pedal edema (DUL pretreatment didn’t decrease the volume of paw edema induced by formalin). However, the ED_50_ between the two studies are different. We think that the different index used to reflect the spontaneous pain behaviors may the important reason for this difference. In the previous study, the flinches time per min was used. Whereas, the pain scores were performed in this investigation, which included favoring, lifting and licking behaviors. The four different doses adopted in these two studies might be another reason for the different ED_50_. This difference might also result from different seasons or environments and age of the two animal groups which can also be observed in the clinical studies and some experiment (Aun et al., 1992; Metzler-Wilson et al., 2013).

### The Expressions of Fos in the SDH and CeA

The immediate early gene *c-fos* is rapidly and transiently expressed in neurons in response to nociceptive stimulation, which encodes for the nuclear protein Fos (Harris, 1998). And levels of the protein peak about 2 h after induction of gene transcription. However, Fos may also contribute to long-term modulation of spinal nociceptive processes is by involvement in the changes in spinal nociceptive circuits that lead to hyperalgesia (increased sensitivity to noxious stimuli) or allodynia (non-noxious stimuli). In this situation, even some touch stimuli would induce nociceptive perception and thus induce the Fos expressions. Lots of studies have shown that expression of Fos in spinal neurons is high following procedures that cause hyperalgesia and allodynia, even last for a long time (Wu et al., 2014; Zhang et al., 2014; Zhao et al., 2017). The CeA, which is known as the “nociceptive amygdala,” receives glutamatergic inputs from the parabrachial nucleus (PB), which convey more than 90% nociceptive information from the spinal dorsal horn in the rodents (Basbaum et al., 2009; Cameron et al., 2015). Lots of neurons in the CeA would be activated after formalin injection being consistent with the previous studies (Zhang et al., 2014; Morland et al., 2016). However, it was reported that intraplantar injection of formalin increased *c-fos* mRNA expression in the BLA, but not CeA (Nakagawa et al., 2003). There were 3 reasons for the difference expressions. Firstly, the Fos protein was checked in the CeA in our study. Maybe, the *c-fos* mRNA expression changes in the CeA was not obvious after intraplantar injection of formalin. Secondly, they detected the *c-fos* mRNA only after formalin injection 1 h. Actually, the expression peak was about 2 h after formalin injection based on the previous investigations (Wu et al., 2014; Yin et al., 2016). At last, the different concentrations of formalin injected into the hindpaw might induce the difference expressions in the CeA.

### The Roles of CeA in the Transition From Acute Pain to Hyperalgesia

The mechanisms underlying the transition from acute spontaneous pain to mechanical and thermal hyperalgesia induced by formalin injection are still unclear. Previous studies reported that formalin could induce secondary allodynia and hyperalgesia, and the intervening measures at SDH, dorsal reticular nucleus, RVM and periaqueductal gray (PAG) all could prevent facilitation of the tail-flick reflex or secondary hyperalgesia after formalin injection (Ambriz-Tututi et al., 2011, 2013; Bravo-Hernandez et al., 2012). It has been demonstrated that pain included not only the somatosensory response, but also the affective response, which was related to the limbic system. But classical studies did not explain the relationship between chronic pain and the negative emotional response very well. The limbic system, especially CeA, took participated in the nociceptive information transmission have been widely confirmed (Zhuo, 2007; Kolber et al., 2010). There is increasing evidences indicating that the limbic system including CeA plays an important role in persistent pain states (Adedoyin et al., 2010; Kolber et al., 2010). Some studies demonstrated that glutamate receptor (mGluR) in the CeA modulated pain-like behavior, moreover pharmacological blockade or conditional deletion of mGluR in the CeA abrogated inflammation-induced diphase hypersensitivity (Adedoyin et al., 2010; Kolber et al., 2010). Whether CeA has influence on the formalin-induced pain transition is still unclear. Our results showed that the CeA was activated during formalin induced spontaneous pain and long-term hyperalgesia. We proposed hypothesis that nociceptive input was sent to LPB firstly via SDH (Traub and Murphy, 2002; Hearn et al., 2014b) and then CeA was activated by LPB (Nakao et al., 2012; Hearn et al., 2014a). The neuroplasticity in the CeA plays a pivotal role in the transition from acute to chronic pain and the initiation of long-term hyperalgesia induced by formalin injection. ACC and insular cortex can also generate long-term potentiation during persistent pain, which received nociceptive information from the CeA (Zhuo, 2007). Is CeA related with ACC or insular cortex in the process of transition from acute pain to long-term hyperalgesia induced by formalin? This will be investigated in our following study.

### The Roles of ERK in the CeA Involved Into the Formalin-Induced Hyperalgesia

ERK is one of important molecule in MAPK signaling pathways, and plays an important role in the inflammatory pain. ERK can be phosphorylated (p-ERK) when noxious substances stimulate sensory neurons (Galan et al., 2003; Ji, 2004). Previous studies have revealed that neuronal ERK activation further increased the activity of TRPV1, which mediated hyperalgesia (Zhuang et al., 2004; Karim et al., 2006). Moreover, the inhibition of ERK signaling pathway is associated with a reduction of hyperalgesia in a neuropathic pain model (Zhuang et al., 2005) and inflammatory pain models (Adwanikar et al., 2004; Kawasaki et al., 2004). Furthermore, the activation of ERK pathway also contributed to pain-related synaptic plasticity in dorsal root ganglion and spinal cord (Kawasaki et al., 2004; Takahashi et al., 2006). Notably, pain-related synaptic plasticity in limbic system was also related to ERK activation (Fu et al., 2008). The activation of ERK in the amygdala was both necessary for and sufficient to induce long-lasting peripheral hypersensitivity to tactile stimulation. Thus, blockade of inflammation-induced ERK activation in the amygdala significantly reduced long-lasting peripheral hypersensitivity associated with persistent inflammation, and pharmacological activation of ERK in the amygdala induced peripheral hypersensitivity in the absence of inflammation (Carrasquillo and Gereau, 2007). Our results were in accordance with these studies. We observed that the activation of ERK in the superficial layers of SDH at 2 h, and in CeA at 2 and 24 h after formalin injection. So we hypotheses that the second-phase acute spontaneous pain behaviors were sustained by the expression of p-ERK in the SDH, but the activation of ERK in the CeA contributed to the formalin-induced transition from acute pain to long-term hyperalgesia after formalin injection. The maintenance of formalin-induced long-term hyperalgesia may also be mediated by the activation of ERK in the CeA after formalin injection 24 h.

## Conclusion

DUL showed an analgesic effect on the spontaneous pain behaviors and hyperalgesia induced by the formalin injection, which may promote the use of DUL on the chronic inflammatory pain. Our study initially found that DUL exert analgesic effect on the long-term hyperalgesia via disrupting the transition from acute pain, which were correlated with the concentration of 5-HT in the CeA. More attention needs to be directed on the limbic system to search new analgesic strategy for the chronic inflammatory pain, as well as the molecular mechanisms mediated this analgesic effect.

## Author Contributions

E-RW and TD: study concept and design. J-BY, LZ, WH, and YS: acquisition of data. Q-RF, Z-CQ, M-JH, and J-BY: analysis and interpretation of data. J-BY, LZ, and E-RW: draft the manuscript. W-JZ, E-RW, and AK: critical revision of the manuscript for important intellectual content. E-RW: study supervision.

## Conflict of Interest Statement

The authors declare that the research was conducted in the absence of any commercial or financial relationships that could be construed as a potential conflict of interest.

## Footnotes

1 www.graphpad.com
