# Supplementary material for: Analgesic Effects of Duloxetine on Formalin-Induced Hyperalgesia and Its Underlying Mechanisms in the CeA
Source: Front Pharmacol. 2018 Apr 10;9:317. doi: 10.3389/fphar.2018.00317 (PMC5902556; doi:10.3389/fphar.2018.00317)
Supplement: Supplementary file 1 [file Data_Sheet_1.docx]

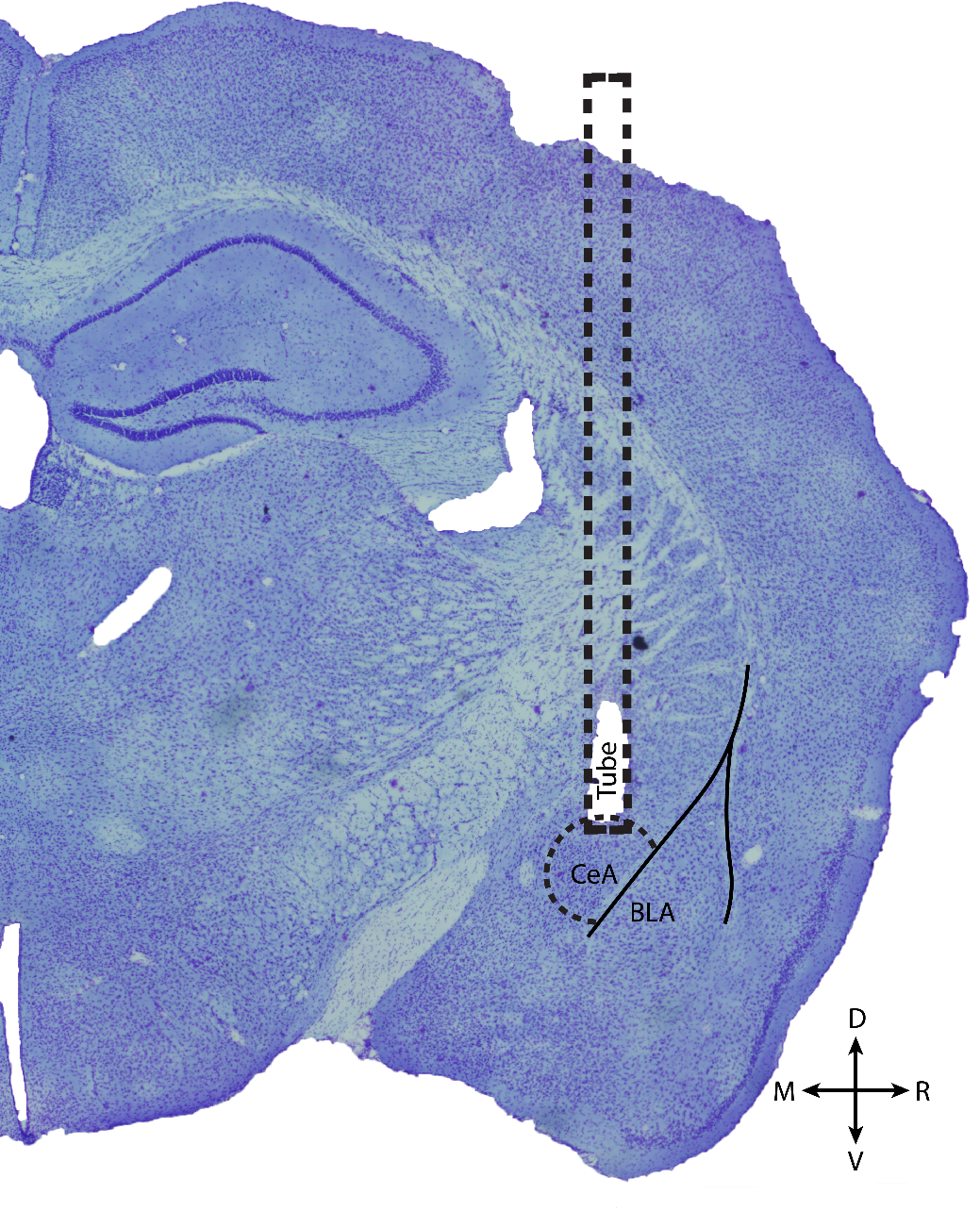


**Supplementary Fig.** The Nissl staining image showed that the cannula was implanted into the CeA (Bregma -1.46 mm). The dashed line above the CeA showed the placement of the cannula. D: dorsal; V: ventral; M: medial; R: right.
